# A machine learning approach for single cell interphase cell cycle staging

**DOI:** 10.1038/s41598-021-98489-5

**Published:** 2021-09-29

**Authors:** Hemaxi Narotamo, Maria Sofia Fernandes, Ana Margarida Moreira, Soraia Melo, Raquel Seruca, Margarida Silveira, João Miguel Sanches

**Affiliations:** 1 Institute for Systems and Robotics (ISR), Instituto Superior Técnico (IST), University of Lisbon, Lisbon, Portugal; 2 Epithelial Interactions in Cancer (EPIC) Group, Instituto de Investigação e Inovação em Saúde (i3S), University of Porto, Porto, Portugal; 3 Institute of Molecular Pathology and Immunology of the University of Porto (IPATIMUP), University of Porto, Porto, Portugal; 4 Institute of Biomedical Sciences Abel Salazar (ICBAS), University of Porto, Porto, Portugal; 5 Faculty of Medicine, University of Porto, Porto, Portugal

**Keywords:** Genomics, Cellular imaging, Image processing

## Abstract

The cell nucleus is a tightly regulated organelle and its architectural structure is dynamically orchestrated to maintain normal cell function. Indeed, fluctuations in nuclear size and shape are known to occur during the cell cycle and alterations in nuclear morphology are also hallmarks of many diseases including cancer. Regrettably, automated reliable tools for cell cycle staging at single cell level using in situ images are still limited. It is therefore urgent to establish accurate strategies combining bioimaging with high-content image analysis for a bona fide classification. In this study we developed a supervised machine learning method for interphase cell cycle staging of individual adherent cells using in situ fluorescence images of nuclei stained with DAPI. A Support Vector Machine (SVM) classifier operated over normalized nuclear features using more than 3500 DAPI stained nuclei. Molecular ground truth labels were obtained by automatic image processing using fluorescent ubiquitination-based cell cycle indicator (Fucci) technology. An average F1-Score of 87.7% was achieved with this framework. Furthermore, the method was validated on distinct cell types reaching recall values higher than 89%. Our method is a robust approach to identify cells in G_1_ or S/G_2_ at the individual level, with implications in research and clinical applications.

## Introduction

The cell cycle is a highly organized and coordinated process that ensures the correct duplication of genetic material and cell division^1,2^. Importantly, the progression of cells through the cell cycle occurs in an orderly sequence of events and encompasses four distinct cell cycle phases termed G_1_ (Gap1), S (synthesis), G_2_ (Gap2) and M phase or mitosis^2,3^. Briefly, in each cell cycle, cells in G_1_ prepare for DNA synthesis, which occurs in the S phase, and subsequently progress to G_2_ to prepare for mitosis^2,3^. All phases are tightly regulated by cell cycle checkpoints, including cyclins and cyclin dependent kinases (CDKs), that control and ensure cells are able to proceed along the cell cycle^1,4^.

Remarkably, dysregulation of the cell cycle occurs in many diseases, such as cancer^1,5^. Indeed, it has been shown that tumor cell cycle dynamics has prognostic value in many cancer types including breast, gastric and prostate cancer^6–8^. Furthermore, cell cycle evaluation has been reported to predict sensitivity or resistance to chemotherapeutic regimens and specific therapeutic strategies for various cancers^9,10^. In addition, cell cycle proteins and regulators have become attractive targets in cancer therapy and novel cell cycle inhibitors have emerged to provide new treatment options for cancer patients^1,11^. Thus, determining cell cycle phases is of critical importance for tumor characterization and monitoring, ultimately impacting cancer care.

To date, most studies on cell cycle staging involve flow cytometry or other methods that often use cells in suspension, analyze cell populations or require specific cell cycle markers and extensive cell manipulation, thus presenting many drawbacks^12^. One example of cell cycle assessment requiring specific cell cycle markers is the recent fluorescent ubiquitination-based cell cycle indicator (Fucci) technology. Fucci is a genetically encoded indicator system of cell cycle progression, in which cells are modified to express cell cycle markers^13–15^. More specifically, the Fucci technology is a fluorescent protein based sensor system that takes advantage of Cdt1 and Geminin, two proteins that oscillate inversely and are involved in the DNA replication control system. These are fused to red and green fluorescent proteins allowing the identification of cells at G_1_ and S/G_2_/M, with nuclei in G_1_ phase red and nuclei in S/G_2_/M phases green^13–15^.

In recent years, novel approaches are emerging taking advantage of nuclear morphological alterations occurring along the cell cycle^16^, thus opening new opportunities for the development of automated bioimaging methods for cell cycle classification. Several strategies based on image processing have been proposed to infer the cell cycle phase of cells based on their DNA content, shape and texture^17–20^. Regrettably, most of the methodologies do not focus on the interphase phases G_1_, S and G_2_, since these are more difficult to classify at cell level or present limitations mainly related to manual parameter tuning^17–22^.

Thus, there is an urgent need to develop automated tools for in situ interphase cell cycle staging of single cells within heterogeneous cell populations as cancer cells, using microscopy images and taking advantage of automated image analysis. In the present work, we propose a combined strategy using deep learning followed by a supervised classifier for interphase cell cycle staging of individual cells using in situ fluorescence images of nuclei stained with the DNA dye 4′,6-diamidino-2-phenylindole (DAPI). Specifically, we present a new pipeline using a deep learning cell nuclei segmentation method^23^ and involving training and testing a machine learning classifier for categorization of nuclei patches from DAPI images in G_1_ or in S/G_2_ classes. Noteworthy, for training, nuclei labels were obtained with molecular biomarkers according to the Fucci technology using Fucci2, a Fucci derivative with different fluorescent properties^13,15^. Subsequently, to further validate our strategy, cell cycle staging was performed on a panel of human gastric, breast and colorectal cancer cell lines. Overall, our data strongly indicates that this method is a robust strategy to successfully identify cells in situ in G_1_ or S/G_2_ at the individual level from distinct cell types, in a rapid and effective manner, which can be applied to heterogeneous cell populations such as cancer cells.

## Results

**Figure 1:**
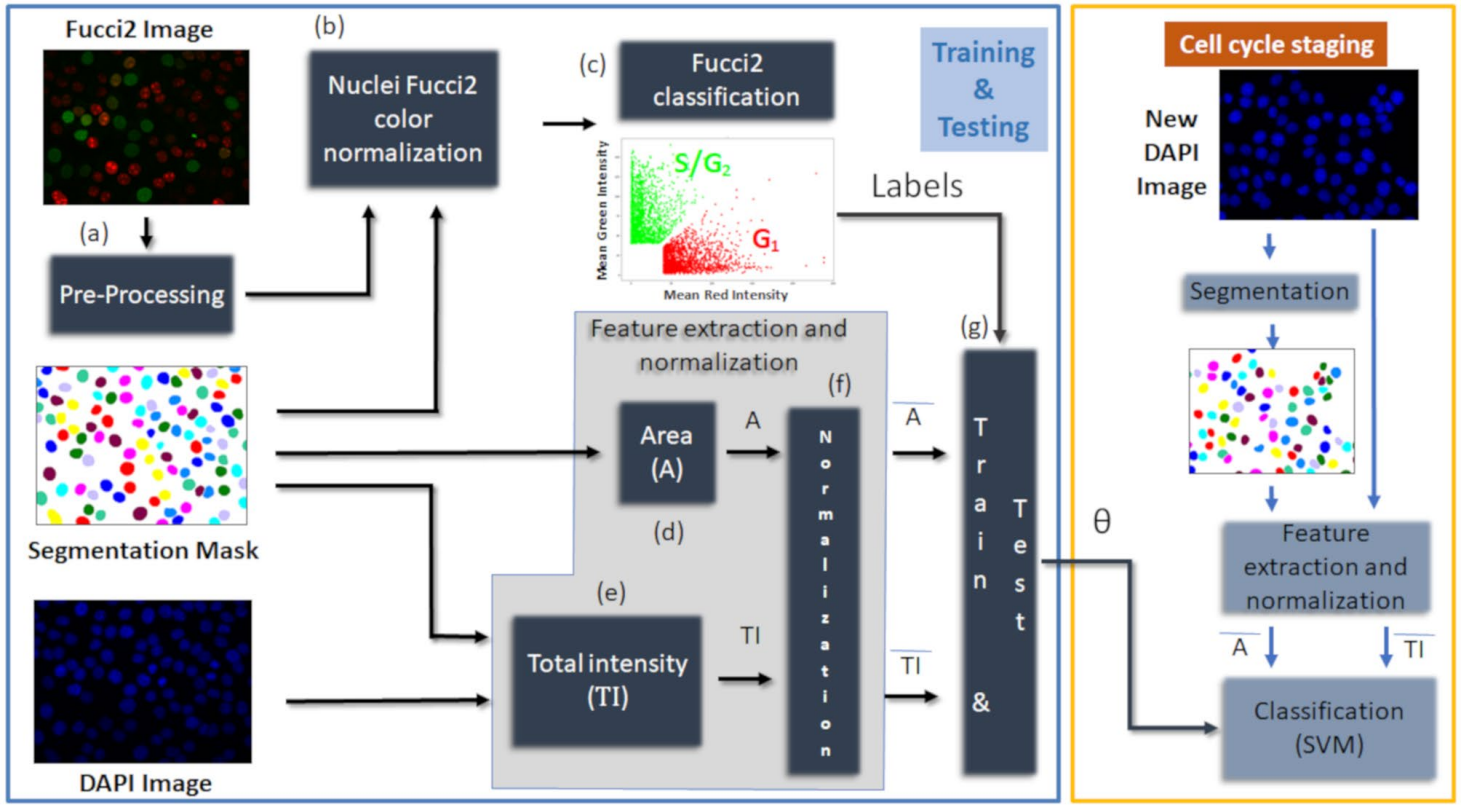
Overall pipeline to train and test the SVM for interphase cell cycle staging. The processing pipeline involves the analysis of DAPI and Fucci2 images, which are required to train and test the classifier (blue box, **a–g**). The final single cell cycle staging procedure uses the classifier parameters, obtained in the training phase, to identify the cell cycle phase of new DAPI images (yellow box); *SVM* support vector machine, *Fucci* fluorescent ubiquitination-based cell cycle indicator.

In this work, we developed a new analytical bioimaging pipeline for interphase cell cycle staging of individual cells using in situ fluorescence images. The strategy involved segmentation, training, cross-validation and testing of a classifier to ensure proper categorization of new data. The overall pipeline is schematically represented in Fig. 1. In particular, the staging is performed by a *Support Vector Machine* (SVM) classifier based on features extracted from nuclei stained with DAPI. The labels, required to train it, are automatically computed from the corresponding image planes of cells expressing Fucci2 fluorescent cell cycle probes using the Fucci technology, available in this dataset for training and performance evaluation. Ultimately, the result of our training procedure is a set of parameters *θ*_*SVM*_, which can be used to classify new DAPI nuclei patches regarding their cell cycle phase based solely on DAPI staining.

As depicted in Fig. 1, training and testing of the SVM classifier included:

1. Fucci2 data processing: (a) image pre-processing; (b) color normalization based on the nuclei segmentation masks and (c) binary automatic classification assuming linearly separable classes in the red-green color space.
2. Features computation: (d) nuclei areas (A) computation from the nuclei segmentation masks, and (e) computation of the total intensity (TI) of nuclei stained with DAPI using the nuclei segmentation masks.
3. Features normalization, training and testing: (f) Features (*A,TI*) normalization, and (g) SVM classifier training and testing using normalized features and labels obtained in (c) from Fucci2 data. The resulting SVM parameters *θ* are used to classify new DAPI nuclei regardless Fucci2 information.

For classification of new DAPI images, nuclei are segmented using the algorithm developed by Narotamo et al.^23^ and features (*A,TI*) are extracted and normalized as in (d), (e) and (f). The classifier parameters obtained in (g), *θ*_*SVM*_, are then used to classify new images.

### Automatic classification of Fucci2 cell cycle labels required for machine learning training

In the present study, a supervised approach for interphase cell cycle staging was developed. For this purpose, supervision and training of the machine learning classifier was performed using cell cycle molecular labels generated based on Fucci technology and automatic image processing (Fig. S1).

#### Fucci2 image processing

In order to train the classifier, processing of the Fucci2 images was necessary. The image processing included an initial pre-processing for background removal, nuclei segmentation, feature extraction and color normalization.

More specifically, a pre-processing framework was applied to Fucci2 images so the intensities of all images were comparable, thus accounting for the variability inherent to immunofluorescence and image acquisition (Fig. S1a). In this step, for each color channel of each image, the mean background intensity was subtracted as described in Materials and Methods. Next, in the segmentation step, we aimed to clearly and efficiently identify and isolate nuclei from the images. A deep learning based segmentation method proposed by Narotamo et al.^23^ was used, in which nuclei were first detected through the Fast YOLO architecture, as detailed in Materials and Methods. The resulting masks, each one containing a single nucleus, were used to compute the labels from the Fucci2 images required for classification (Fig. S1b). Finally, for feature extraction and color normalization, the features from the *k*th nucleus were computed from the original images by multiplying DAPI, *D*(*i, j*) or Fucci2, [*F*^*r*^(*i, j*)*,F*^*g*^(*i, j*)] by the binary mask associated with that nucleus, *b*_*k*_(*i, j*), obtained in the segmentation step. The resulting nucleus specific intensity images are *d*_*k*_(*i, j*) = *b*_*k*_(*i, j*)*D*(*i, j*) and [*f*_*k*_^*r*^(*i, j*)*, f*_*k*_^*g*^(*i, j*)] = *b*_*k*_(*i, j*)[*F*^*r*^(*i, j*)*,F*^*g*^(*i, j*)]. Finally, for all the other nuclei, the extracted features were computed according to the Eqs. (1)–(4) (Table 1). Based on these features, additional parameters were computed according to the Eqs. (5)–(8) (Table 1).

**Table 1:** Intensity and morphological features equations.

Feature	Equation	
Area: number of pixels in the nucleus	${A}_{k}= \sum_{i,j}{b}_{k}(i,j)$	(1)
Total DAPI intensity: sum of the intensities of the pixels in the nucleus extracted from the DAPI image	${TI}_{k}^{b}=\sum_{i,j}{b}_{k}\left(i,j\right)D(i,j)$	(2)
Total red intensity: sum of the red channel’s intensities of the pixels in the nucleus extracted from the Fucci2 image	${TI}_{k}^{r}=\sum_{i,j}{b}_{k}\left(i,j\right){F}^{r}(i,j)$	(3)
Total green intensity: sum of the green channel’s intensities of the pixels in the nucleus extracted from the Fucci2 image	${TI}_{k}^{g}=\sum_{i,j}{b}_{k}\left(i,j\right){F}^{g}(i,j)$	(4)
Mean red intensity	${\mu }_{k}^{r}=\frac{{TI}_{k}^{r}}{{A}_{k}}$	(5)
Mean green intensity	${\mu }_{k}^{g}=\frac{{TI}_{k}^{g}}{{A}_{k}}$	(6)
Normalized red intensity: to capture the red color independently of the intensity	${\overline{\mu }}_{k}^{r}=\frac{{\mu }_{k}^{r}}{\sqrt{{\left({\mu }_{k}^{r}\right)}^{2}+{\left({\mu }_{k}^{g}\right)}^{2}}}$	(7)
Normalized green intensity: to capture the green color independently of the intensity	${\overline{\mu }}_{k}^{g}=\frac{{\mu }_{k}^{g}}{\sqrt{{\left({\mu }_{k}^{r}\right)}^{2}+{\left({\mu }_{k}^{g}\right)}^{2}}}$	(8)

#### Establishment of Labels from Fucci2 Data

**Figure 2:**
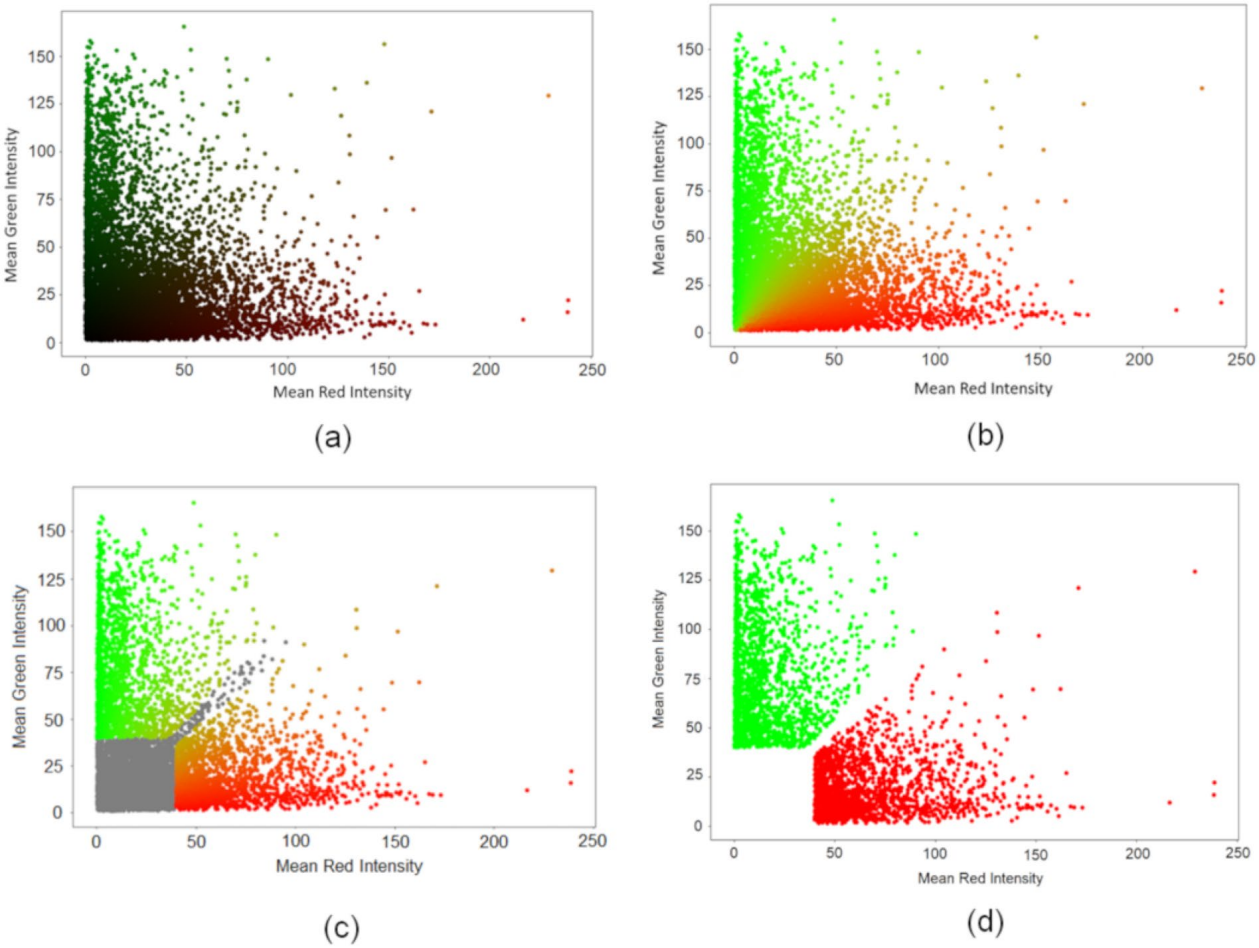
Nuclei distribution in the 2D space [*µ*_*k*_^*r*^*,µ*_*k*_^*g*^] and strategy to automatically estimate the labels from the Fucci2 data to train the supervised classifier. **(a)** Each point is colored with the intensities (R,G,B) = [*µ*_*k*_^*r*^*,µ*_*k*_^*g*^*,*0]. **(b)** Each point is colored with the intensities (R, G, B) =$\left[{\overline{\mu }}_{k}^{r}, {\overline{\mu }}_{k}^{g},0\right]$_*k*_*.* **(c)** The nuclei that were excluded are colored in grey. All the other nuclei are colored with the following intensities (R, G, B) = $\left[{\overline{\mu }}_{k}^{r}, {\overline{\mu }}_{k}^{g},0\right]$. **(d)** Representation of each nucleus in the 2D space [*µ*_*k*_^*r*^*,µ*_*k*_^*g*^] after removing the colorless nuclei, outliers, and some nuclei in the transition between red and green. Each nucleus is represented by a single colored dot in the 2D space. Each point is colored with its label, i.e., nuclei labeled as S/G_2_ are green, whereas nuclei labeled as G_1_ are red.

Fucci2 data was used to automatically compute cell cycle labels of each nucleus required to train the classifier. Each *k*th nucleus is represented by a single colored dot in the two-dimensional (2D) space of red–green (RG) colors by its mean intensities [*µ*_*k*_^*r*^*,µ*_*k*_^*g*^], according to Eqs. (5) and (6) as shown in Fig. 2a. The RGB color of each dot is [*µ*_*k*_^*r*^*,µ*_*k*_^*g*^*,*0]. Normalized values are represented in Fig. 2b, in which the color of each dot is computed using the Eqs. (7) and (8) to improve the ability to discriminate the three classes G_1_, S and G_2_. The goal of this normalization is to obtain the true color of each dot independently of its intensity. It is the main added value of the proposed automatic approach for label classification when compared with the manual, in which nuclei true color identification is difficult when intensities are low.

In Fig. 2b, we can observe each nucleus with the normalized intensity and the unit slope straight line (represented in blue) that separates G_1_ and G_2_ according to Fucci2 technology. At transition, the technique is not conclusive because cells are undergoing from G_1_ to S. The normalization procedure is able to enhance color differences between both classes by normalizing to 1 the norm of the color vector of each nucleus $\left[{\overline{\mu }}_{k}^{r}, {\overline{\mu }}_{k}^{g}\right]=\frac{\left[{\mu }_{k}^{r},{\mu }_{k}^{g}\right]}{\sqrt{{\left({\mu }_{k}^{r}\right)}^{2}+{\left({\mu }_{k}^{g}\right)}^{2}}}$ and low intensity nuclei become clearly visible. However, since no additional information was added, the confidence on these nuclei is still reduced.

Thus, low intensity nuclei and nuclei at the transitions or aberrant areas were excluded from the analysis as shown in Fig. 2c (in grey) according to the following criteria, in which the parameters were chosen in a trial and error basis:

1. $${\mu }_{k}^{r}$$*,*$${\mu }_{k}^{g}$$ <*ε*, too dark (*ε* = 40)
2. τ_1_ < ${\mu }_{k}^{g}$*/*${\mu }_{k}^{r}$ <τ_2_, too similar (τ_1_ = 0.9 and τ_2_ = 1.1)
3. |A − *µ*_*A*_|> *σ*_*A*_, too large or too small areas.

The first inequality selects colorless nuclei and the second nuclei at the transition region. The third condition rejects nuclei with aberrant sizes where *µ*_*A*_ and *σ*_*A*_ are the mean and standard deviation of nuclei areas (A), respectively. The resulting nuclei used to train the classifier are displayed in Fig. 2d and the respective labels were obtained according to Algorithm 1:

**Table:** 

Algorithm 1: Automatic algorithm to assign a label to each nucleus based on molecular features.
1: if ${\mu }_{k}^{g}$ >${\mu }_{k}^{r}$ then label = S/G_2_
2: if ${\mu }_{k}^{r}$ >${\mu }_{k}^{g}$ then label = G_1_

In this work, a total of 3553 nuclei were considered for analysis, with 2291 nuclei (64.5%) labeled as G_1_ and 1262 nuclei (35.5%) labeled as S/G_2_.

### Comparison of cell cycle labels obtained automatically and by visual inspection

**Figure 3:**
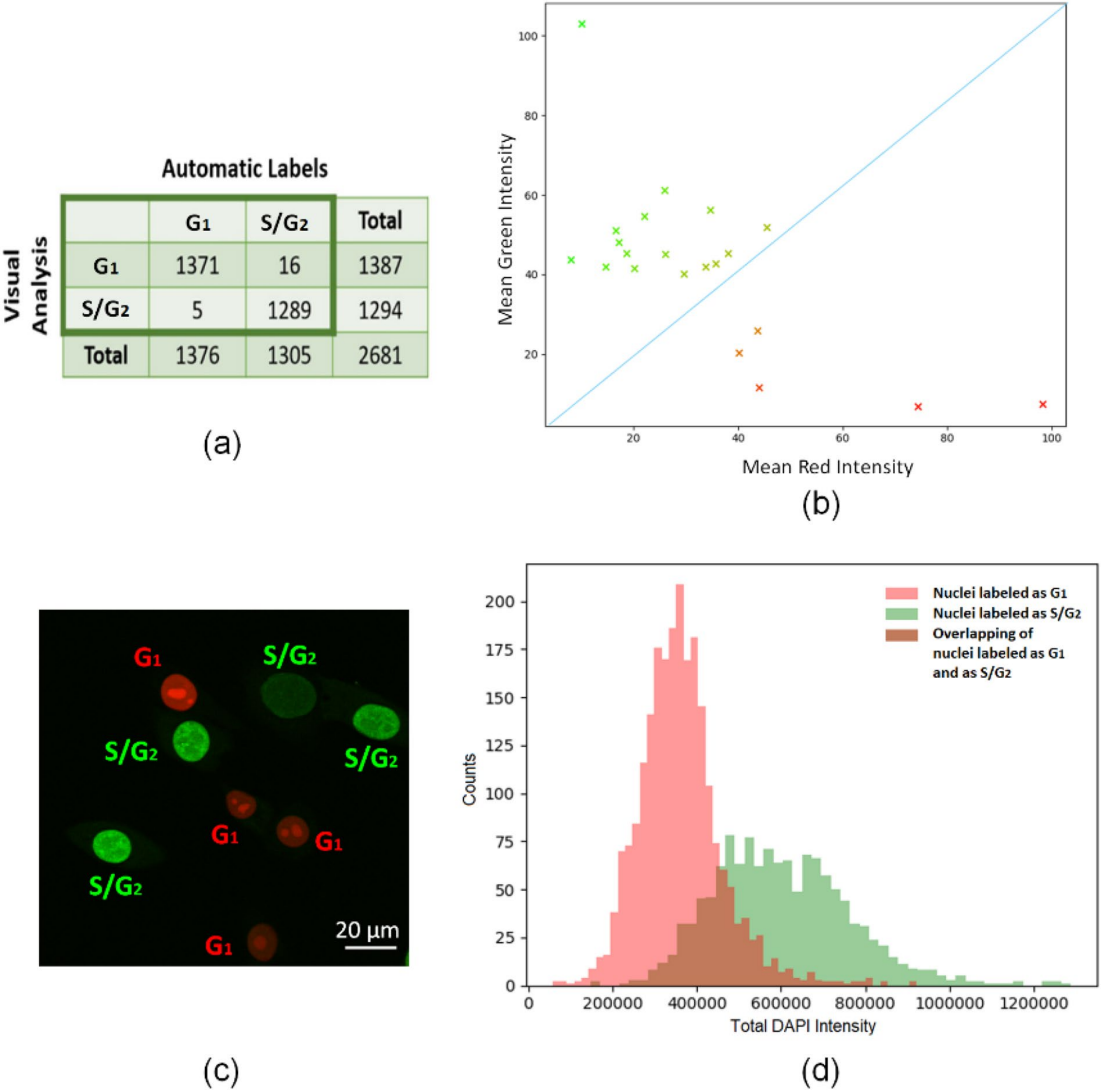
Identification of labels obtained automatically and by visual inspection. **(a)** Confusion matrix between the labels generated according to Algorithm 1 and by visual analysis. **(b)** Representation of each nucleus, labeled by visual analysis, in the 2D space $[{\mu }_{k}^{r},{\mu }_{k}^{g}]$. Each point is colored with the following intensities (R, G, B) = $\left[{\overline{\mu }}_{k}^{r}, {\overline{\mu }}_{k}^{g},0\right]$. The blue line corresponds to the equation mean green intensity = mean red intensity, and is used to identify mislabeled nuclei evaluated by visual analysis. **(c)** Example of a Fucci2 image and the corresponding manual labels obtained by visual analysis. **(d)** Cell cycle profile of total DAPI intensity for labeled nuclei. The red and green bars indicate counts of nuclei labeled as G_1_ and S/G_2_, respectively. The brown area shows the overlapping bars of nuclei labeled as G_1_ and as S/G_2_.

In this study, we have developed an automatic procedure to generate cell cycle labels obtained from Fucci2 data, required to subsequently train the classifier. This approach aimed to avoid difficulties associated with interpretation of Fucci2 images by visual inspection. Specifically, these difficulties can occur upon (i) low intensity (dark) nuclei; (ii) transition states (G_1_ to S) due to co-expression of cell cycle reporters; or (iii) non-homogeneous cases in which nuclei may exhibit different colors in different nuclear structures. Thus, to overcome this limitation, an automatic strategy was proposed. Herein, the clustering results obtained automatically using Algorithm 1 (*A*_1_) were compared with the labels obtained by visual analysis (VA) based on the Fucci technology. Of note that only nuclei classified by VA were considered for further analysis, which did not include all nuclei classified by *A*_1_. The results indicate that from a total of 2681 nuclei, only 21 nuclei diverged in the classification, corresponding to an accuracy of 99*.*22%. Specifically, as shown in Figs. 3a, 5 nuclei automatically labeled by *A*_1_ as G_1_ were labeled as S/G_2_ by VA, and 16 nuclei labeled by the *A*_1_ as S/G_2_ were labeled by VA as G_1_. The high accuracy obtained is explained by the fact that VA only considered nuclei that could be clearly classified. Figure 3b displays the 21 nuclei labeled differently by *A*_1_ and VA, with the 16 nuclei above the blue line labeled by VA as G_1_ and the 5 nuclei below this line labeled by VA as S/G_2_. Importantly, the blue line represents the bisectrix of the first quadrant that roughly separates G_1_ and G_2_ classes. An example of a Fucci2 image and the corresponding labels obtained by VA is shown in Fig. 3c. The divergences between the automatic method of label detection and the ground truth provided by VA are possibly due to misclassifications related to color miss-perception inherent to the human operator that the automatic method solved after color normalization. Overall, these results suggest that the automatic proposed method has higher accuracy than the manual approach using visual inspection and is much less time consuming, thus improving our strategy.

### Automatic cell cycle staging and comparison between Fucci2 labels and DAPI features

Our strategy to analyze the interphase cell cycle stages takes advantage of the common DNA binding dye DAPI. In this work, in order to further evaluate nuclei labeled by Algorithm 1, a cell cycle profile was generated from total DAPI intensity, as shown in Fig. 3d. The histogram, which was obtained from the distribution of labeled nuclei within the distinct phases, reveals a higher number of nuclei in G_1_ and higher DNA content in S/G_2_ than in G_1_ (Fig. 3d), which is consistent with a typical cell cycle phase distribution. Overall, these results support the use of our automatic approach in the generation of cell cycle labels.

**Figure 4:**
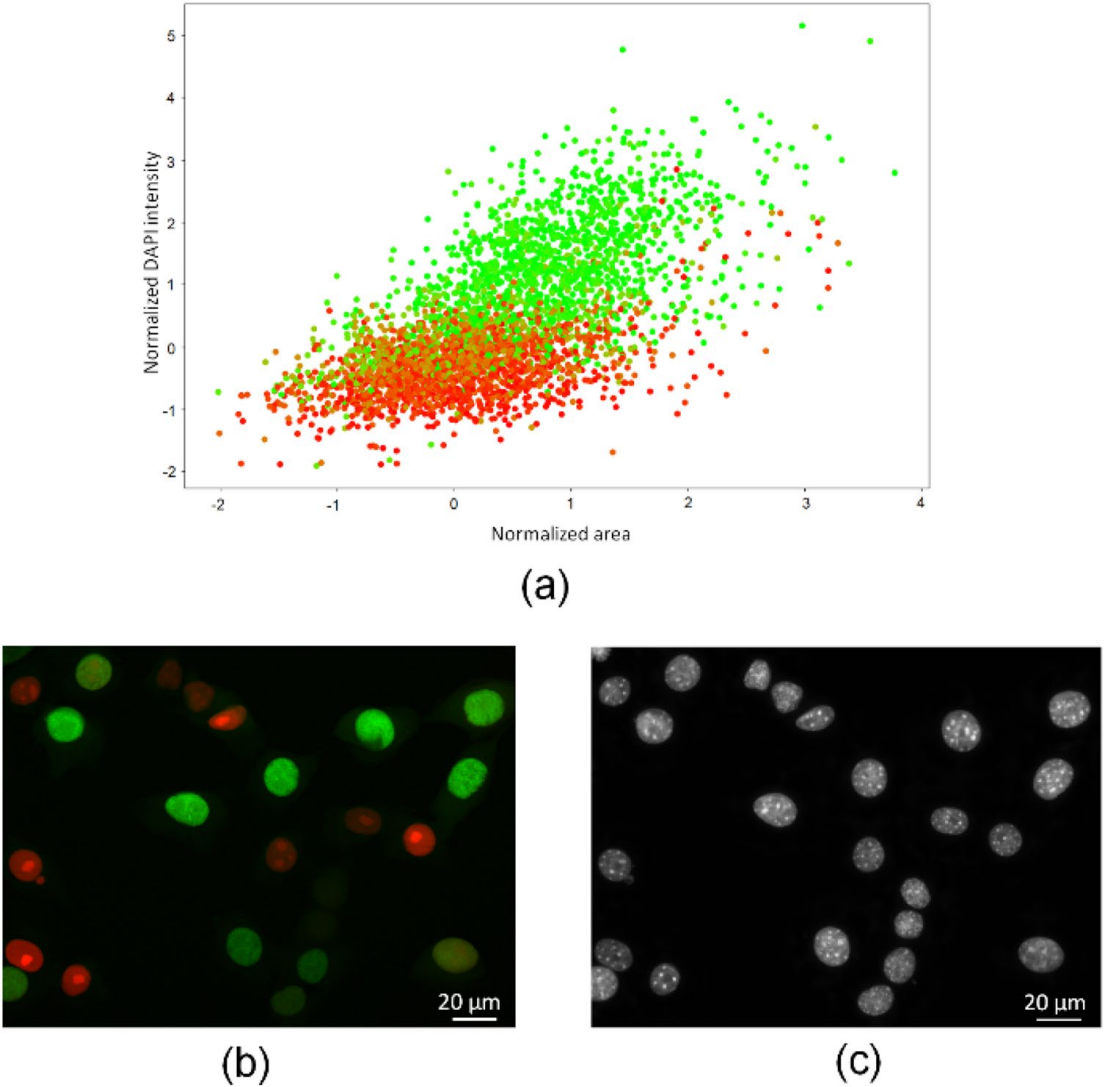
Comparison between Fucci2 labels and DAPI features. Representation of each nucleus in the 2D space (normalized area, normalized DAPI intensity). Each point is colored with the following intensities (R, G, B) = $\left[{\overline{\mu }}_{k}^{r}, {\overline{\mu }}_{k}^{g},0\right]$, according to the Fucci2 classification. **(b,c)** Representative Fucci2 and DAPI images respectively, illustrating the brightness and size of nuclei in S/G_2_ and G_1_ stages.

Moreover, in order to further study the association between Fucci2 and DAPI data, we have represented the 3553 labeled nuclei in the 2D space (normalized area, normalized DAPI intensity). As shown in Fig. 4, the results demonstrate that green nuclei in S/G_2_, as determined by Fucci technology, exhibit higher DAPI intensity and larger area than red nuclei in G_1_. Taken together, these results corroborate our previous data^21^ and strongly support the variations in DNA content along the cell cycle, which correlate with DAPI staining. In summary, our results confirmed that the information obtained from the Fucci technology regarding the cell cycle can also be obtained from DAPI nuclear staining.

### Nuclei classification based on DAPI features and performance evaluation

Our results indicate that DAPI area and intensity can be used to evaluate the cell cycle. Therefore, a SVM was trained and tested with DAPI features as input, and the ground truth labels were generated from Fucci2 images.

Normalized features, ${\overline{f} }_{i}$ were used to train the SVM classifier. These features are zero mean and unit variance normalized versions of the ones in Eqs. (1) (area) and (2) (DAPI intensity), computed from DAPI image planes, according to:

9 $${\overline{f} }_{i}=\frac{{f}_{i}-{\mu }_{f}}{{\sigma }_{f}},$$

where *µ*_*f*_ and *σ*_*f*_ are the mean and the standard deviation corresponding to feature *f*_*i*_, computed for each image separately.

Finally, we assessed cell cycle staging on new DAPI images. Firstly, nuclei segmentation was performed and features were extracted from DAPI images (intensity) and from the corresponding segmentation masks (area). Thereafter, these features were normalized according to Eq. (9), by computing *µ*_*f*_ and *σ*_*f*_ for nuclei area and intensity. These normalized features were then fed to the classifier that returned the cell cycle phase (G_1_ or S/G_2_) for each nucleus.

Nuclei classification was subsequently validated and the estimation of prediction error was performed using both nested five-fold cross-validation and nested leave-one-experiment-out cross-validation.

#### Nested five-fold cross-validation

The performance of the nuclei classification strategy was evaluated using Precision, Recall and F1-Score, as shown in Table 2. In this table, data represent Precision, Recall and F1-Score when class G_1_ or class S/G_2_ were considered positive, as well as the average between both classes. The values correspond to the mean and standard deviation over the five models obtained by performing nested five-fold cross-validation.

**Table 2:** Validation of nuclei classification (µ ± σ).

	Precision	Recall	F1-Score
G_1_	0*.*904 ± 0*.*021	0*.*926 ± 0*.*015	0*.*915 ± 0*.*006
S/G_2_	0*.*858 ± 0*.*025	0*.*822 ± 0*.*040	0*.*839 ± 0*.*019
Average	0*.*881 ± 0*.*016	0*.*874 ± 0*.*022	0*.*877 ± 0*.*010

Precision, Recall and F1-Score data are shown for class G_1_ positive, class S/G_2_ positive and average between both classes. Data represent mean ± standard deviation over the five models obtained by performing nested five-fold cross-validation.

As shown in Table 2, the proposed approach for cell cycle staging can be performed based on features extracted from DAPI images, as demonstrated by high Precision, Recall and F1-Scores. Specifically, the results demonstrate that the critical features to be considered are relative area and DAPI intensity, since nuclei in S/G_2_ phases have their area increased and present higher DAPI intensity when compared to nuclei in G_1_ phase.

Importantly, in this study, the features from all nuclei, from all images were used to train the SVM. The SVM was trained and tested in the normalized input space, with features from different images, which resulted in high F1-scores (F1-Scores 0*.*915 ± 0*.*006 and 0*.*839 ± 0*.*019 for class G_1_ and class S/G_2_, respectively). This result can be explained by the fact that this SVM takes into account the relative area and intensity for each nucleus. Thus, this procedure is computationally less demanding when compared to other approaches. Indeed, although we perform feature normalization per image, it only takes about 12 s to compute the normalized area and intensity according to Eq. (9) for all the nuclei analyzed. Overall, our data indicate that the SVM presented in this work can take as input nuclei features from all images to perform cell cycle staging providing an accurate label for each nucleus.

Notably, as observed in Fig. 4a, the most challenging issue is to classify nuclei in G_1_ and S/G_2_ that have overlapping intensities. Thus, to further evaluate our approach, we have analyzed the performance of our method in nuclei with normalized DAPI intensities ranging from -0.5 to 0.5. This subset of data included 525 nuclei, corresponding to approximately 15% of our data set. Precision, Recall and F1-Score obtained for this subset were 0.72, 0.77 and 0.74, respectively. Therefore, even for this challenging subset, our approach was able to achieve an F1-Score of 74%.

#### Nested leave-one-experiment-out cross-validation

**Figure 5:**
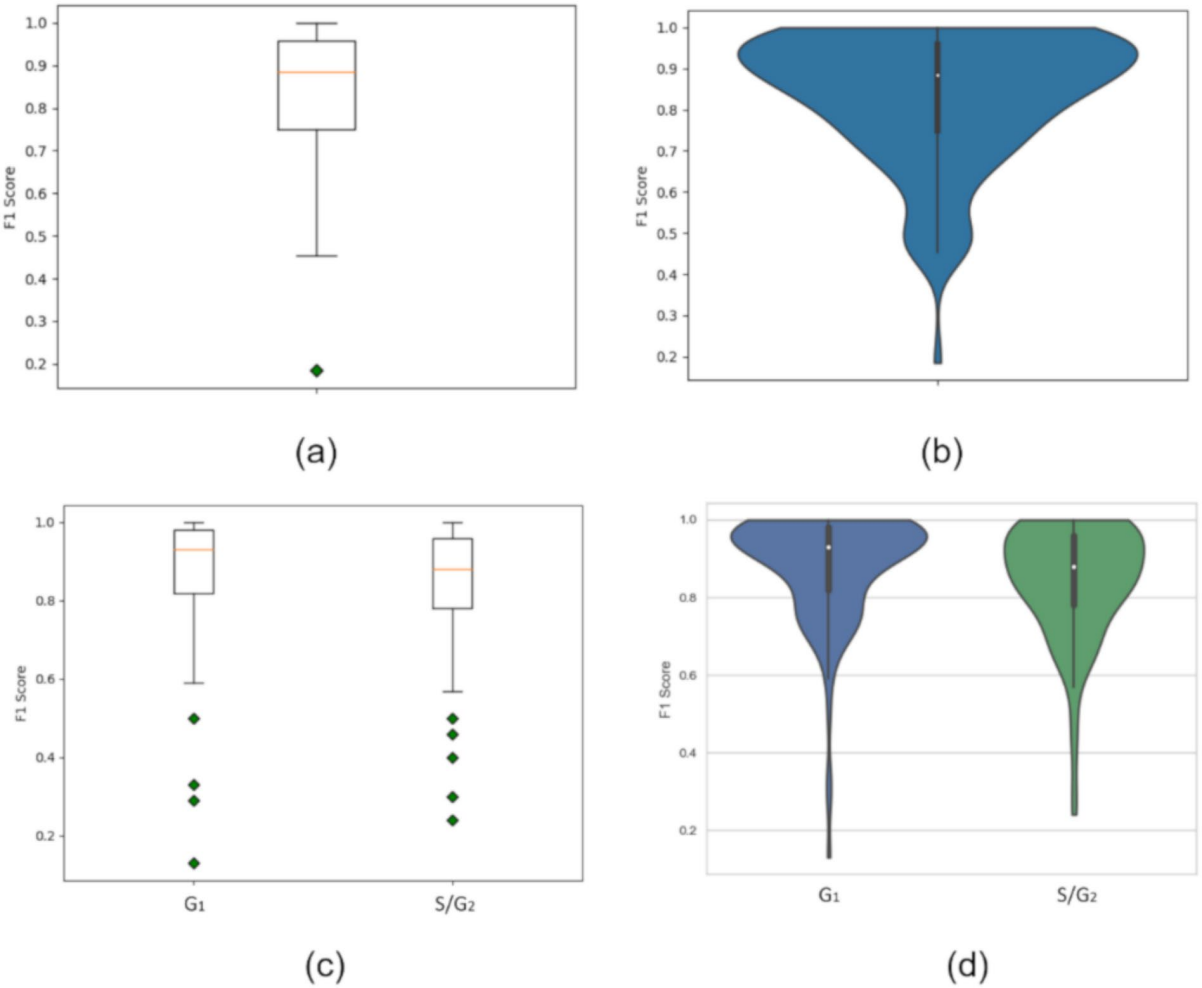
Distribution of nuclei classification **(a)** box plot of F1-Score (130 images). **(b)** Violin plot of F1-Score (130 images). **(c)** Box plot of F1-Score for class G_1_ and class S/G_2_. **(d)** Violin plot of F1-Score for class G_1_ and for class S/G_2_.

Next, to further assess our strategy, a nested leave-one-experiment-out cross-validation was performed. More specifically, we conducted a nested 13-fold cross-validation to understand how the feature normalization step influences the performance of the proposed approach in each image. A total of 130 images were analyzed and the corresponding 130 values of average F1-Score between class G_1_ and S/G_2_ are represented in Fig. 5. Remarkably, in 21 of the 130 images, the classifier assigned the correct class to all nuclei (average F1-Score of 100%). Furthermore, as observed in Fig. 5a,b, the distribution of the data is not symmetric. Indeed, in 91 images a high F1-Score was obtained, which was equal or higher than 80%. Moreover, as observed in Fig. 5b, most of the values are clustered around the maximum, that is, the peak with the highest amplitude is located at an F1-Score of approximately 93%. These results show that, for most of the images, our classifier provides proper classification results.

Noteworthy, in this study, feature normalization was performed considering that variations in image acquisition can occur even using the same acquisition parameters. More specifically, area and intensity normalization were conducted for each image and through this normalization step, the relative area and intensity between nuclei in G_1_ phase and nuclei in S/G_2_ phases were obtained. However, in images with all nuclei at a particular stage, the normalization step could only provide the relative area and intensity of the nuclei at that particular stage, and no information regarding nuclei at the other stage could be made available. Interestingly, the few images with nuclei in one of the classes, corresponded to the images for which an F1-Score lower than 50% was obtained, as observed in Fig. 5b. Moreover, in Fig. 5a, we can detect the presence of an outlier that corresponds to an image with 1 nucleus in G_1_ and 15 nuclei in S/G_2_. An alternative strategy to avoid this problem, is to perform the normalization per experiment rather than per image, to guarantee enough nuclei in G_1_ and in S/G_2_. Still, this normalization step may also be affected by acquisition differences between the images.

In addition, box and violin plots of F1-Score for class G_1_ and class S/G_2_ are shown in Fig. 5b,d. Notably, the data demonstrate better results for class G_1_ than those obtained for class S/G_2_ (Fig. 5d). This can be explained by the fact that our dataset is imbalanced. As previously described in Fig. 5a, the outliers in Fig. 5b correspond to images that have the majority or all nuclei in one of the two possible classes. By examining the violin plots in Fig. 5d, it can be concluded that for about 75% of the images the F1-Scores for class G_1_ are higher than 80% and for about 75% of the images, the F1-Scores for class S/G_2_ are higher than 78%. Importantly, for both classes the maximum F1-Score obtained is 100%.

### Validation of the classifier on distinct cell types

**Figure 6:**
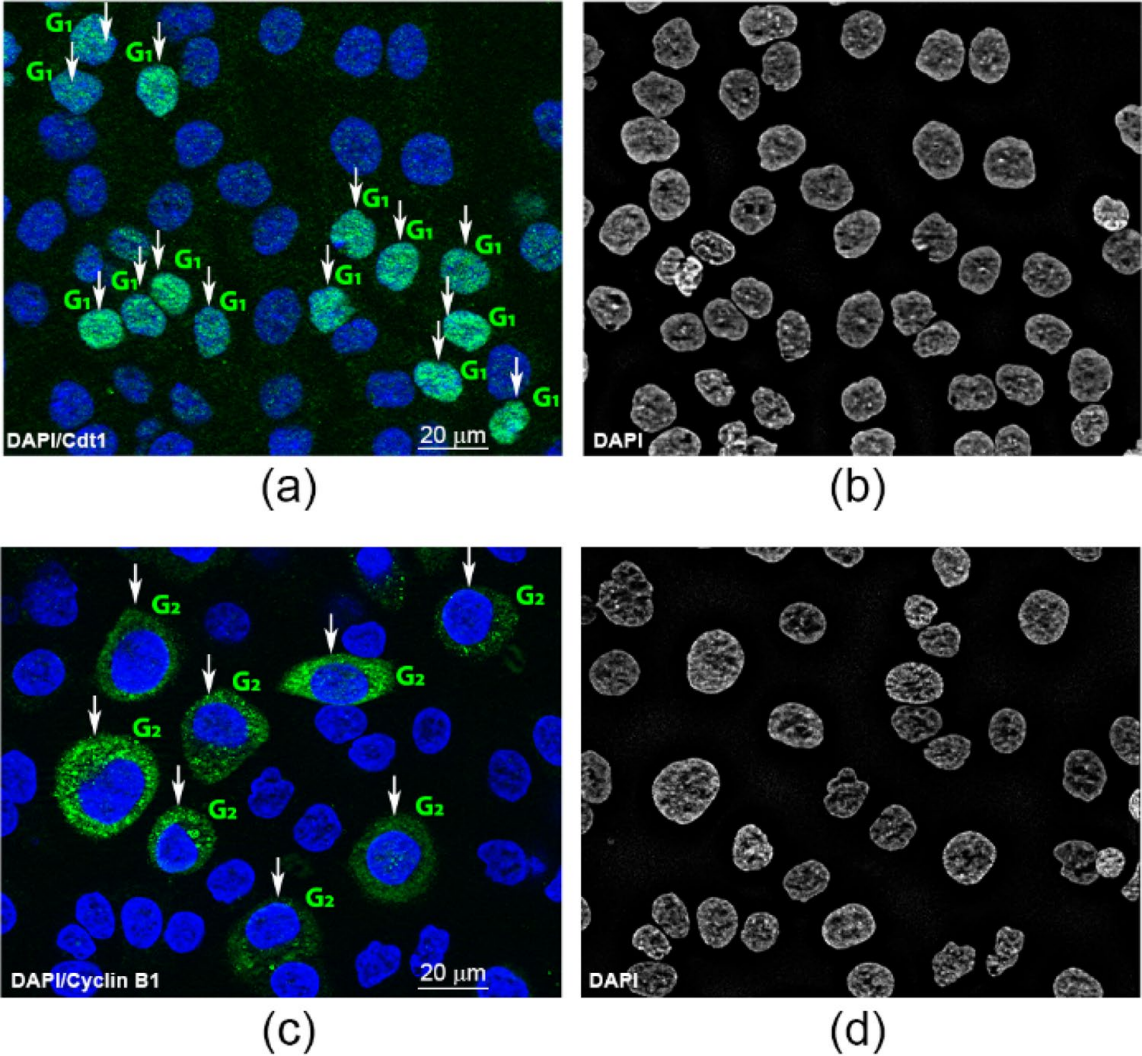
Method applicability in immunofluorescence images of gastric cancer cells. Human gastric cancer cells AGS were stained with Cdt1 (green) **(a)** or cyclin B1 (green) **(c)** and counterstained with DAPI (blue). White arrows indicate cells considered positive for Cdt1 or cyclin B1 expression and used for further analysis and G_1_ and G_2_ denotes the labels obtained after automatic classification based on DAPI staining. **(b,d)** Corresponding DAPI images used for cell cycle staging.

In order to study the potential of the SVM for cell cycle staging in different cell types, we evaluated a panel of human gastric (AGS, MKN74), breast (MDA-MB-231, MCF7) and colorectal cancer (SW480, HCT116) cell lines, distinct from the cell type used to train it. Specifically, the SVM was trained with all nuclei (3553 nuclei) and its performance was tested on images of human gastric, breast and colorectal cancer cells. Cells were stained with DAPI for cell cycle staging and with Cdt1 or cyclin B1 for validation purposes. An example is shown in Fig. 6, with Cdt1 positive cells labelled in green and nuclear DAPI in blue (Fig. 6a). Similarly, Fig. 6c illustrates cyclin B1 positive cells labelled in green and nuclear DAPI in blue. The corresponding DAPI images used for cell cycle staging are presented in Fig. 6b and Fig. 6d. Nuclei in G_1_ and G_2_ were annotated manually based on the expression of Cdt1 or cyclin B1, markers of G_1_ and G_2_/M phases, respectively. Note that mitotic nuclei were not considered in this study and therefore nuclei likely to be at the M phase were excluded. Moreover, only nuclei with strong Cdt1 and cyclin B1 fluorescence intensity were selected for analysis, as ascertained by visual inspection. This dataset included 180 images, comprising 15 images for each cell type, for each specific antibody. For analysis, nuclei were segmented, features area and DAPI total intensity were computed and normalized, and each segmented nucleus was classified using the SVM previously trained. Interestingly, high recall values (ranging from 89.1 to 99.5%) were obtained for all cell lines analyzed. Specifically, recall values were as follow: AGS (Cdt1: 93.8%; cyclin B1: 93.3%), MKN74 (Cdt1: 99.5%; cyclin B1: 93.2%), MDA-MB-231 (Cdt1: 90.1%; cyclin B1: 89.1%), MCF7 (Cdt1: 89,6%; cyclin B1: 91.8%), SW480 (Cdt1: 94.3%; cyclin B1: 96.1%), HCT116 (Cdt1: 91.1%; cyclin B1: 96.9%). Taken together, the data strongly support that this method can be used for cell cycle staging of distinct cell types.

## Discussion

Cell cycle evaluation has long been a prime issue in biological and cancer research and the focus of many clinical studies. However, and despite technical advances, cell cycle staging remains challenging, namely using automated methods in non-manipulated cells and for single cell analysis. Over the years, extensive efforts have been made to improve available approaches but most methods still rely on the evaluation of cell populations and involve the use of cell cycle markers and extensive cell manipulation that disrupt the natural cellular architecture^12^. Importantly, both limitations account for the lack of applications in minimal invasive methodologies in cancer diagnosis and monitoring, as cytology and CTCs analysis. Recently, novel strategies have emerged involving automated imaging analysis in the field of computational life sciences. Distinct frameworks have been proposed in recent years^17,19,22,24–27^. Still, most of the available procedures are not suitable for in situ analysis and interphase cell cycle staging at single cell level.

In this study, we propose a new pipeline for interphase cell cycle staging of single cells that is based on nuclear features using in situ cell images and that can be applied to heterogeneous cell populations. In particular, we developed a new bioimaging pipeline using in situ fluorescence images of nuclei stained solely with DAPI, involving a deep learning segmentation procedure followed by a supervised classifier (SVM) for phase identification. In order to establish the SVM, training was achieved using molecular ground truth labels. Indeed, for training purposes, we have specifically developed an automatic procedure to identify cell cycle labels based on Fucci data to overcome the limitations associated with classification by visual inspection. Our results have shown that the automatic method is more accurate and less time consuming than the manual approach, which are two fundamental properties in transferring new technologies into clinical applications or to be translated into innovation platforms for high throughput drug discovery.

Notably, our strategy takes advantage of the extensively used DNA dye DAPI that exhibits strong fluorescence when bound to adenine–thymine rich sequences of DNA and that correlates with DNA content along the cell cycle^12,28^. Noteworthy, our data has shown a typical cell cycle histogram following automatic cell cycle staging based on DAPI, further supporting our approach. Moreover, DAPI area and intensity predicted cell cycle staging, in a simple and easier strategy, while avoiding the use of cell cycle markers and laboratory manipulation. Remarkably, high Precision, Recall and F1-score were achieved upon performance evaluation. In addition, our classifier was further validated on a panel of six distinct human cancer cells types including gastric, breast and colorectal cancer cells, solely stained with DAPI. Cell cycle regulatory proteins are expressed during particular phases of the cell cycle and are often used to identify specific phases, as Cdt1^13,15^ and cyclin B1^11–13,15^. Thus, in order to obtain the ground truths, cells were stained with DAPI and Cdt1 or cyclin B1, specific cell cycle markers that were used to annotate nuclei in G_1_ or G_2_, respectively. The results demonstrate that the SVM was able to properly classify nuclei in G_1_ and G_2_ based on DAPI, independently of the cell type. Remarkably, our approach was able to achieve high recall values for all cell lines, despite different nuclear sizes specific to each cell line, highlighting the importance of the feature normalization step proposed in this work. Therefore, although the SVM was trained with features from murine mammary gland cells stained with DAPI, the SVM can also be used for cell cycle staging of other cell types.

When compared to previously described methods, our cell cycle staging pipeline holds advantages. For instance, in the method developed by Roukos et al.^22^ the validation step involved comparison of the cell cycle distributions with the ones obtained by flow cytometry^22^. In contrast, in this work, a nucleus by nucleus validation was performed and for each nucleus the predicted G_1_ or S/G_2_ cell cycle phase was compared with the ground truth label of that nucleus. Moreover, in the protocol proposed by Roukos et al. only one DAPI feature was used, the integrated intensity, which depends on the experimental conditions^22^. Furthermore, although the method allows cell cycle staging of individual cells, it is based on manually defined thresholds, which may be subjective^22^. Hence, compared to the work presented by Roukos et al*.*^22^, the proposed approach has the advantage of using automatic methods for cell cycle staging. In addition, our former framework, also presents some limitations. In that approach, area and total DAPI intensity were calculated for each nucleus, which did not result from automatic nuclei segmentation, and the obtained features formed the feature space in which a modified K-means clustering was performed achieving an overall sensitivity of 94%^21^. However, it relied on features that may vary between different experiments and different cell lines, and thus the K-means algorithm needed to be applied image by image^21^. Thus, our previous method^21^ is more time-consuming compared to the proposed approach. Furthermore, in our previous work^21^ we did not study the performance of the method on other cell lines and therefore cannot conclude about its generalization capability. Importantly, the major drawback of both the above mentioned methods is the nuclei segmentation step, since in both cases it relies on traditional nuclei segmentation methods which can be sensitive to noise and depend on manual parameter tuning^29^. In contrast, we used a deep learning based segmentation method in which nuclei were first detected through the Fast YOLO architecture^23^. Moreover, in our previous work, the ground truth labels generated to validate the performance of the method resulted from an intensive work of the observer with inevitable pitfalls^21^. In addition to these, other data analysis workflows are becoming available including DeepFlow^26^. However, a major drawback of this approach is the lower accuracy in detecting interphase cells (79.40%) as compared to cells in mitosis (98.73%), which demonstrates the difficulty to distinguish cells at interphase. Furthermore, this technique alters the natural architecture of adherent cells, since it requires cells in suspension^26^, and therefore it is not suitable for cell cycle staging of adherent cells in situ. One of the most challenging issues in cell cycle staging is the classification of G_1_ and S/G_2_ nuclei with overlapping intensities. However, even for this nuclei subset, our approach achieved similar results obtained by others^26^. In the future, additional features may be extracted, such as textural features, to further distinguish this subset of nuclei.

Overall, our data strongly suggest that this method is cell independent, reliable and robust and that in the future it could be used to evaluate single cells within cell populations, which can clearly have an impact in cancer research and clinical applications. Ultimately, we anticipate the proposed pipeline can have major implications in disease monitoring, in the development of novel cell cycle inhibitors in large scale screens, as well as in the selection of appropriate therapeutic strategies along disease progression namely using CTCs analysis.

## Materials and methods

This section describes in detail the supervised approach used for interphase cell cycle staging and the datasets required for testing and training the classifier.

### Datasets

In this study, two distinct datasets were used to test, train and validate the classifier. The first includes data obtained as previously described by Ferro et al*.* and comprises a set of 130 fluorescence microscopy images, from a panel of thirteen datasets of images (corresponding to eight independent cell passages), with more than 3500 nuclei with a resolution of 1040 × 1388 pixels^21^. This is the same dataset used by Ferro et al., in which a non-supervised approach for cell-cycle staging was proposed^21^. The images are from murine mammary gland NMuMG-Fucci2 cells (RCB2868, RIKEN Cell Bank, Japan) constitutively expressing Fucci2 probes and stained with DAPI. Briefly, cells cultured on glass coverslips were fixed and stained with DAPI and images were acquired using the acquisition settings described in^21^. These are organized as RGB images with Fucci2 data on the red and green channels and DAPI information on the blue channel. An additional set of 180 images from human gastric (AGS, MKN74), breast (MDA-MB-231, MCF7) and colorectal (SW480, HCT116) cancer cells, stained with DAPI and Cdt1 or cyclin B1 as described in Materials and Methods, was also used to experimentally validate the proposed method. This dataset included 30 images of each cell type, 15 labeled with Cdt1 and 15 with cyclin B1. The use of a completely different set of images as input to the classifier was performed for generalization ability assessment.

### Fucci2 image processing and image segmentation

All Fucci2 images were subject to an image processing pipeline in order to automatically compute the labels for the classifier. For background removal, a pre-processing framework was applied to all images. Briefly, for each color channel of each image, the mean background intensity was subtracted. For each image the ground truth nuclei segmentation mask was obtained from manual annotation. Based on the mask for each image, the average background’s intensity for each color channel was calculated and denoted as ($\overline{\mathrm{R} },\overline{\mathrm{G} },\overline{\mathrm{B} }$)_background_. Thereafter, every pixel in the image was obtained by subtracting to its intensity ((R,G,B)_pixel_) the average background’s intensity per channel. That is, by computing the following quantity: (R,G,B)_pixel_ – ($\overline{\mathrm{R} },\overline{\mathrm{G} },\overline{\mathrm{B} }$)_background_. Subsequently, nuclei segmentation was performed for all images. Specifically, we have used our recently developed deep learning based segmentation method, in which nuclei are first detected in the images using the Fast YOLO architecture^23^. Afterwards, patches corresponding to the detected nuclei were extracted from the image, resized to a fixed size and used as input of a U-Net, which will compute the corresponding segmentation mask of the patch that is resized back to its original size^23^. Notably, nuclei at the borders were not considered for analysis, since the provided information may be incomplete. The segmentation experiments were carried out on a NVIDIA GPU GTX 1050 (4 GB) and Python 3.6. The deep learning implementations were based on the open-source deep learning libraries Tensorflow and Keras. The segmentation model described in this section is available at https://github.com/HemaxiN/YOLO_UNET. Training of the deep learning approach for Fucci2 images was performed as described in our previous work^23^. For these images, a F1-Score above 0.8 was obtained for IoU thresholds below 0.75. To segment the DAPI images, the model trained in^23^ was used.

### Nuclei classification from DAPI features

For nuclei classification, normalized DAPI features were used to train the SVM classifier^30^. Thus we trained and evaluated the performance of the SVM in the 2D input space of normalized area and normalized DAPI intensity. Hyperparameter optimization was performed in the following parameter space:

- Kernel: 'rbf’; Gamma: (1e−2, 1e−3, 1e−4, 1e−5); C: (0.001, 0.10, 0.1, 10, 25, 50, 100, 1000);
- Kernel: 'poly’; C: (0.001, 0.10, 0.1, 10, 25, 50, 100, 1000); degree: (1, 2, 3, 4, 5);
- Kernel: 'sigmoid’; Gamma: (1e−2, 1e−3, 1e−4, 1e−5); C: (0.001, 0.10, 0.1, 10, 25, 50, 100, 1000);
- Kernel: 'linear’; C: (0.001, 0.10, 0.1, 10, 25, 50, 100, 1000);

To perform the nested five-fold cross-validation the entire dataset was divided into five folds (A, B, C, D and E). The training and testing of the model was performed five times, considering each of the folds (A, B, C, D and E) as test set once. A schematic representation of the five-fold cross-validation is shown in Supplementary Fig. S2. For instance, considering fold E as test set, a model was trained using folds A, B, C and D. The training/validation split is 80%/20%, and for each parameter combination, a model was built using the training set and its performance was evaluated on the validation set. The best model is the one that presents the highest score (average F1-Score between G_1_ and S/G_2_) on the validation set. Finally, the performance of this model was evaluated on the test set (fold E in this example). This procedure was repeated five times (considering folds A, B, C, D and E as test set at a time), and the final results represent the average ± standard deviation of the five test folds.

Furthermore, we also performed a nested 13-fold cross-validation since the dataset includes images from 13 experiments, equivalent to a nested leave-one-experiment-out cross-validation. That is, the model was trained with nuclei from 12 experiments, and its performance was tested on nuclei from the other experiment. This process was repeated 13 times. The average F1-Score values between class G_1_ and S*/*G_2_ were represented in box and violin plots in order to understand the distribution of the data. As stated before, the features of the proposed input space were normalized image by image.

Additionally, for both experiments, class weights were set inversely proportional to the class frequencies in the training data. These weights are inversely proportional to the class frequencies in the training data:

10 $$cweight\_i=\frac{nsamples}{nclasses x nsamples\_i}$$

where cweight_i denotes the class weight for the class i, nsamples the number of samples in the training data, nclasses the number of classes in the classification problem, and nsamples_i the number of samples belonging to class i. This is a technique used to deal with imbalanced datasets, and it will train the SVM in training samples with different cost weights in the objective function^31^. Herein, there are more nuclei in G_1_ phase than in S*/*G_2_ phases, and therefore the class weight is higher for S*/*G_2_ nuclei. The classification experiments were carried out in Python 3.6 using the SVM implementation available in scikit learn (version 0.21.2). The cell cycle staging algorithm developed in this work is available at https://github.com/HemaxiN/InterphaseCellCycleStaging.

### Cell culture, immunofluorescence staining and image acquisition

Human gastric (AGS, MKN74), breast (MDA-MB-231, MCF7) and colorectal (SW480, HCT116) cancer cells were obtained from the American Type Culture Collection (ATCC). Briefly, cells were grown in RPMI 1640 (AGS, MKN74, SW480, HCT116), DMEM (MDA-MB-231) or DMEM/F-12 (MCF7) (Gibco, Invitrogen) supplemented with 10% fetal bovine serum (Hyclone) and 1% penicillin/ streptomycin (Gibco, Invitrogen) in a humidified incubator at 37 °C, 5% CO_2_. Cells were cultured on glass coverslips and fixed with 4% paraformaldehyde for 20 min. Following a 10 min wash in phosphate buffered saline (PBS), cells were permeabilized with 0.1% Triton X-100 in PBS for 15 min at room temperature. Cells were blocked with 3% bovine serum albumin (BSA) in PBS and stained overnight at 4 °C with Cdt1 rabbit primary antibody (1:200, Cell Signaling, #8064) or cyclin B1 rabbit primary antibody (1:800, Cell Signaling #12231). Subsequently, cells were incubated with Alexa Fluor 488 goat anti-rabbit (1:200, Invitrogen, Thermo Fisher Scientific) for 1 h in the dark. Nuclei were stained with DAPI (Sigma-Aldrich, 0.1 µg/ml in PBS) for 15 min and coverslips were mounted on slides using Vectashield (Vector Laboratories). Images were acquired on a Carl Zeiss Apotome Axiovert 200 M Fluorescence Microscope (Carl Zeiss, Jena, Germany) with a 40× objective (Plan-Apochromat 40x/1.3 Oil DIC (UV) VIS-IR M27) using an Axiocam HRm camera and the Zeiss Axion Vision 4.8 software. For DAPI staining, multiple images were acquired along the z axis (60 z stacks; 5 ms exposure) and images were deconvoluted using deconvolution express in Huygens Software (Scientific Volume Imaging). All images were acquired with the same acquisition settings. For Cdt1 and cyclin B1 staining, a single plane image was acquired to confirm cells in the G_1_ or G_2_ phase of the cell cycle, respectively. Image J software was used for analysis.

## Supplementary Information

Supplementary Figures.

## References

[1] Otto T, Sicinski P (2017). Cell cycle proteins as promising targets in cancer therapy. Nat. Rev. Cancer.

[2] Nurse P (2000). A long twentieth century of the cell cycle and beyond. Cell.

[3] Norbury C, Nurse P (1992). Animal cell cycles and their control. Annu. Rev. Biochem..

[4] Vermeulen K, Van Bockstaele DR, Berneman ZN (2003). The cell cycle: A review of regulation, deregulation and therapeutic targets in cancer. Cell Prolif..

[5] Malumbres M, Barbacid M (2009). Cell cycle, CDKs and cancer: A changing paradigm. Nat. Rev. Cancer.

[6] Loddo M (2009). Cell-cycle-phase progression analysis identifies unique phenotypes of major prognostic and predictive significance in breast cancer. Br. J. Cancer.

[7] Sommariva S, Tarricone R, Lazzeri M, Ricciardi W, Montorsi F (2016). Prognostic value of the cell cycle progression score in patients with prostate cancer: A systematic review and meta-analysis. Eur. Urol..

[8] Begnami MD, Fregnani JH, Nonogaki S, Soares FA (2010). Evaluation of cell cycle protein expression in gastric cancer: Cyclin B1 expression and its prognostic implication. Hum. Pathol..

[9] Dokumcu K, Farahani RM (2019). Evolution of resistance in cancer: A cell cycle perspective. Front. Oncol..

[10] Hallett RM (2015). Treatment-induced cell cycle kinetics dictate tumor response to chemotherapy. Oncotarget.

[11] Sherr CJ, Bartek J (2017). Cell cycle-targeted cancer therapies. Annu. Rev. Cancer Biol..

[12] Eastman AE, Guo S (2020). The palette of techniques for cell cycle analysis. FEBS Lett..

[13] Sakaue-Sawano A (2008). Visualizing spatiotemporal dynamics of multicellular cell-cycle progression. Cell.

[14] Sakaue-Sawano A, Kobayashi T, Ohtawa K, Miyawaki A (2011). Drug-induced cell cycle modulation leading to cell-cycle arrest, nuclear mis-segregation, or endoreplication. BMC Cell Biol..

[15] Sakaue-Sawano A, Miyawaki A (2014). Visualizing spatiotemporal dynamics of multicellular cell-cycle progressions with fucci technology. Cold Spring Harb. Protoc..

[16] Jevtic P, Edens LJ, Vukovic LD, Levy DL (2014). Sizing and shaping the nucleus: Mechanisms and significance. Curr. Opin. Cell Biol..

[17] Blasi T (2016). Label-free cell cycle analysis for high-throughput imaging flow cytometry. Nat. Commun..

[18] Chen X, Zhou X, Wong ST (2006). Automated segmentation, classification, and tracking of cancer cell nuclei in time-lapse microscopy. IEEE Trans. Biomed. Eng..

[19] Wang M (2008). Novel cell segmentation and online SVM for cell cycle phase identification in automated microscopy. Bioinformatics.

[20] Yan, J. *et al.* An effective system for optical microscopy cell image segmentation, tracking and cell phase identification. In *IEEE International Conference on Image Processing, ICIP; 1917–1920.*10.1109/ICIP.2006.313143 (2006).

[21] Ferro A (2017). Blue intensity matters for cell cycle profiling in fluorescence DAPI-stained images. Lab. Invest..

[22] Roukos V, Pegoraro G, Voss TC, Misteli T (2015). Cell cycle staging of individual cells by fluorescence microscopy. Nat. Protoc..

[23] Narotamo, H., Sanches, J. M. & Silveira, M. Segmentation of cell nuclei in fluorescence microscopy images using deep learning. in* Pattern Recognition and Image Analysis. IbPRIA 2019. Lecture Notes in Computer Science*. Vol. 11867. 53–64. (Morales A., Fierrez J., Sánchez J., Ribeiro B. eds). 10.1007/978-3-030-31332-6_5 (Springer, 2019).

[24] Mao Y, Han L, Yin Z (2019). Cell mitosis event analysis in phase contrast microscopy images using deep learning. Med. Image Anal..

[25] Li F, Zhou X, Ma J, Wong ST (2010). Multiple nuclei tracking using integer programming for quantitative cancer cell cycle analysis. IEEE Trans. Med. Imaging.

[26] Eulenberg P (2017). Reconstructing cell cycle and disease progression using deep learning. Nat. Commun..

[27] Gomes CJ, Harman MW, Centuori SM, Wolgemuth CW, Martinez JD (2018). Measuring DNA content in live cells by fluorescence microscopy. Cell Div..

[28] Kapuscinski J (1995). DAPI: A DNA-specific fluorescent probe. Biotech. Histochem..

[29] Xing F, Yang L (2016). Robust nucleus/cell detection and segmentation in digital pathology and microscopy images: A comprehensive review. IEEE Rev. Biomed. Eng..

[30] Cortes C, Vapnik V (1995). Support-vector networks. Mach. Learn..

[31] Y.-M., H. & S.-X., D. Weighted support vector machine for classification with uneven training class sizes. *Int. Conf. Mach. Learn. Cybern. (Guangzhou, China)* **7**, 4365–4369. 10.1109/ICMLC.2005.1527706 (2005).

