# Supplementary material for: A machine learning approach for single cell interphase cell cycle staging
Source: Sci Rep. 2021 Sep 29;11:19278. doi: 10.1038/s41598-021-98489-5 (PMC8481278; doi:10.1038/s41598-021-98489-5)
Supplement: Supplementary file 1 — Supplementary Figures. [file 41598_2021_98489_MOESM1_ESM.docx]

**Supplementary Material**


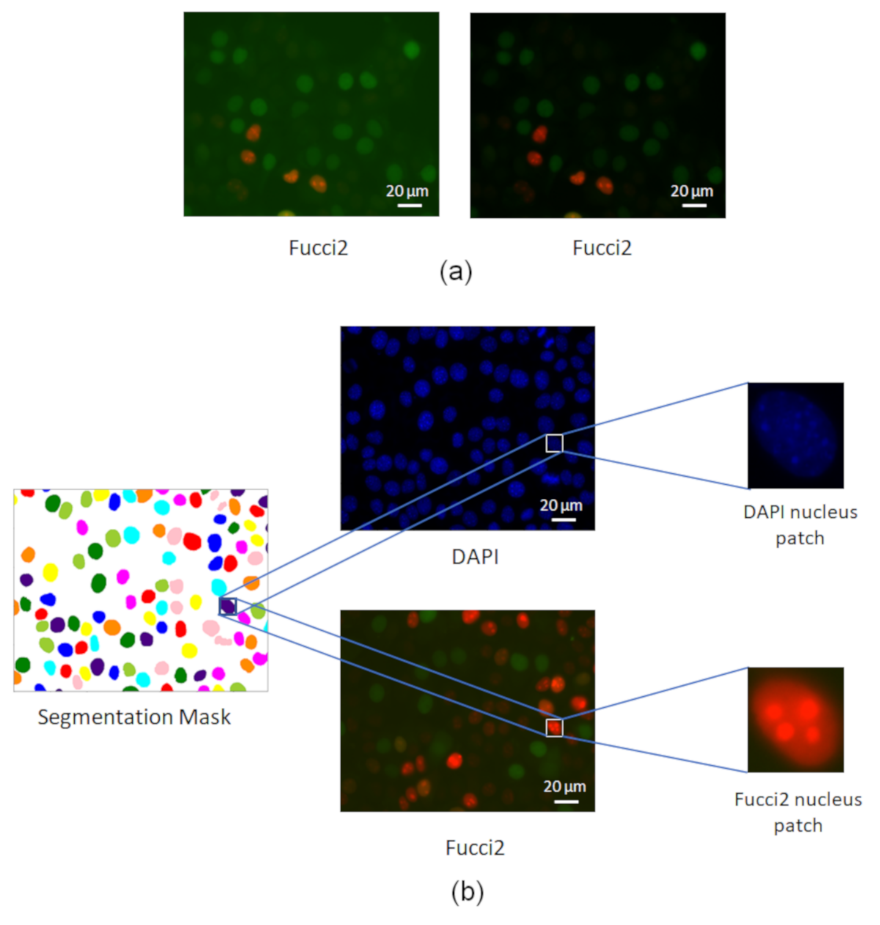


Supplementary Figure S1 - Overview of image processing and nuclear patch extraction for nuclear features computation. (a) Effect of background removal as illustrated in the Fucci2 image before processing (left) and after background removal (right). (b) Schematic representation of nuclear patch extraction from DAPI and Fucci2 images based on the segmentation mask obtained after applying the deep learning based approach. Nuclei patches were extracted from original images with a resolution of 1040x1388 pixels.


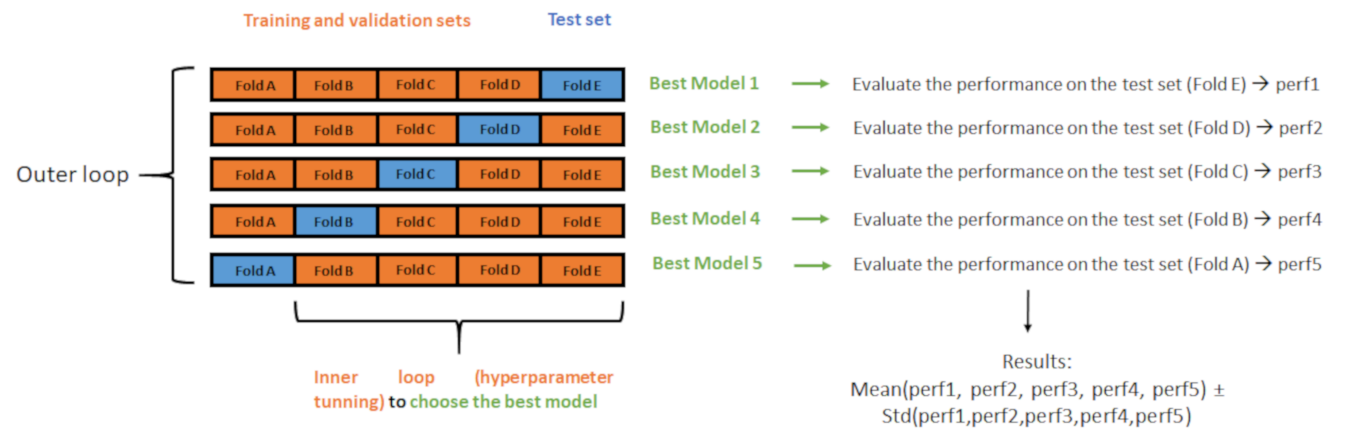


Supplementary Figure S2 – Schematic representation of the nested five-fold cross validation The nested five-fold cross-validation was performed by dividing the dataset into five folds. In the inner loop the training/validation split is 80%/20%. In this loop 112 models (for each parameter combination) are trained using the training set. Their performance (perf) is evaluated one by one on the validation set. Afterwards, the model with the best performance on the validation set is used to evaluate its performance on the test set. The results correspond to the mean ± standard deviation of the performance in the five test sets.
